# Genetic variants in *ERCC1* and *XPC* predict survival outcome of non-small cell lung cancer patients treated with platinum-based therapy

**DOI:** 10.1038/s41598-017-10800-5

**Published:** 2017-09-06

**Authors:** Ruoxin Zhang, Ming Jia, Huijing Xue, Yuan Xu, Mengyun Wang, Meiling Zhu, Menghong Sun, Jianhua Chang, Qingyi Wei

**Affiliations:** 1 Cancer Institute, Collaborative Innovative Center for Cancer Medicine, Fudan University Cancer Center, 270 Dong’an Road, Xuhui, Shanghai, 200032 China; 2 Department of Oncology, Shanghai Medical College, Fudan University Shanghai Cancer Center, 270 Dong’an Road, Shanghai, 200032 China; 3 School of Medicine, Shanghai Jiaotong University, 227 South Chongqing Road, Shanghai, 200025 China; 4 Department of Oncology, Xinhua Hospital affiliated to Shanghai Jiaotong University, No. 1665 Kong Jiang Road, Shanghai, 200092 China; 5 Department of Pathology, Fudan University Shanghai Cancer Center, 270 Dong’an Road, Shanghai, 200032 China; 6 Department of Medical Oncology, Fudan University Shanghai Cancer Center, 270 Dong’an Road, Shanghai, 200032 China; 7 Duke Cancer Institute, Duke University Medical Center, 10 Bryn Searle Dr., Durham, NC 27710 USA

## Abstract

Nucleotide excision repair (NER) plays a vital role in platinum-induced DNA damage during chemotherapy. We hypothesize that regulatory single nucleotide polymorphisms (rSNPs) of the core NER genes modulate clinical outcome of patients with advanced non-small cell lung cancer (NSCLC) treated with platinum-based chemotherapy (PBS). We investigated associations of 25 rSNPs in eight NER genes with progression free survival (PFS) and overall survival (OS) in 710 NSCLC patients. We found that *ERCC1* rs3212924 AG/GG and *XPC* rs2229090 GC/CC genotypes were associated with patients’ PFS (HR_adj_ = 1.21, 95% CI = 1.03–1.43, *P*_adj_ = 0.021 for *ERCC1* and HR_adj_ = 0.80, 95% CI = 0.68–0.94, *P*_adj_ = 0.007 for *XPC*), compared with the AA and GG genotypes, respectively. The association of *XPC* rs2229090 was more apparent in adenocarcinoma than in squamous cell carcinoma patients. Additionally, *ERCC4* rs1799798 GA/AA genotypes were associated with poorer OS (HR_adj_ = 1.32, 95% CI = 1.04–1.69, *P*_adj_ = 0.026), compared with the GG genotype. The expression quantitative trait loci analysis revealed that *ERCC1* rs3212924 and *XPC* rs2229090 might regulate transcription of their genes, which is consistent with their associations with survival. Larger studies are needed to validate our findings with further functional studies to elucidate the mechanisms underlying these observed associations.

## Introduction

Lung cancer is the leading cause of cancer-related deaths worldwide, accounting for an estimation of 1.59 million deaths in 2012^1^, while China attributed to 529,153 deaths in 2011^2^. Non-small cell lung cancer (NSCLC) patients, representing approximately 85% of the total lung cancer incident cases, are often diagnosed at an advanced stage of IIIB or IV^3, 4^. Because surgical treatment for these advanced-stage patients is not possible, palliative chemotherapy remains the mainstream therapeutic option. The overall five-year survival for lung cancer has remained less than 15%, and the prognosis for the advanced-stage disease is even poorer, with a median overall survival (OS) of approximately one year^5, 6^.

Platinum-based regimes are the standard first-line chemotherapy for NSCLC patients, although recent targeted therapies have presented benefits for a small portion of the patients who have activating *EGFR* mutations or *EML-ALK* translocations^7, 8^. However, the efficacy of platinum-based chemotherapy (PBC) varies remarkably among the patients, with an overall response rate of 26~60%^9^. It is speculated that this may be related to individual variability in repairing DNA damage induced by PBC^10, 11^. Increasing body of evidence highlights the importance of genetic factors, such as single nucleotide polymorphisms (SNPs), and gene expression in individual response to the treatment, which have an impact on subsequent survival^12^, particularly for genetic variations in nucleotide excision repair (NER) genes^13–16^.

The DNA repair pathways are the safeguard of genomic stability by restoring damaged DNA induced by mutagens (i.e. UV, tobacco or chemicals), of which NER is the major mechanism removing bulky DNA lesions caused by chemicals. NER has been frequently associated with survival in NSCLC patients treated with PBC^13^. NER functions by repairing platinum-DNA (Plt-DNA) adducts, involving the coordination of 20–30 proteins that replace the bulky adduct DNA segment with a newly synthesized DNA segment using the intact complementary strand as the template^17^. The hypotheses of NER genes affecting lung cancer prognosis are two-folds, a double edged sword: on one side, suboptimal DNA repair may promote carcinogenesis by weakening mutation-fixation of DNA damage induced by both exogenous and endogenous carcinogens and subsequent development of tumours^18^ and the other side, efficient DNA repair in the tumour may lead to fast removal of plt-DNA adducts, reducing the efficacy of PBC^13, 19^.

NER comprises of three main events: recognition of base damage, the bimodal incision of DNA, and excision of DNA fragments^17, 20, 21^. The specific recognition of substrate sites consists of several key proteins: the initial step involves the XPC-HHRAD23 complex, which recognizes the base damage caused by exogenous carcinogens^22^. The XPE/DDB1 protein has been studied for its auxiliary role for the recognition of cyclobutane pyrimidine lesions, due to its affinity for UV-damaged DNA^23^. The XPC/HHRAD23 complex further binds to several other proteins (i.e. XPA, RPA, TFIIH and XPG/ERCC5), in which transcription factor IIH (TFIIH) is a subcomplex of the RNA polymerase II transcription initiation machinery, and XPB/ERCC3 and XPD/ERCC2 are two central DNA helicases that unwind the DNA duplex in the close vicinity of the base damage; XPG and ERCC1-XPF heterodimeric protein are two endonucleases that cut the damaged DNA strand 3′ and 5′ to the site of the base damage, respectively^20, 22^. These core proteins work in concert to maintain NER function, and hence their respective roles in the NER pathway have been more extensively studied.

In the present study, we undertook a hypothesis-based approach to evaluate the impact of regulatory SNPs (rSNPs) in the core NER genes (*ERCC1, ERCC2, ERCC3, ERCC4, ERCC5, RAD23B, XPA, XPC* and *XPE*) on survival of NSCLC patients treated with PBC by analysing a pool of 25 rSNPs in 710 patients with advanced disease stages. All these 25 rSNPs were predicted by bioinformatics tools to be potentially functional in regulating their gene expression (Table 1 and Supplemental S1).

## Results

### Characteristics of the study population

The present study consisted of 710 patients diagnosed with NSCLC^24^, who had DNA samples, complete data on demographic, clinical characteristics, progression free survival (PFS) and overall survival (OS). Of all the patients, 508 were males and 202 were females, with a median age at diagnosis of 58 (a range of 23–83) years, and 334 (47%) were never, 41 (5.8%) former, and 335 (47.2%) current smokers. All subjects had an advanced TNM stage (III or IV) cancer, with 478 (67.3%) being adenocarcinoma. For different chemotherapy combinations, 237 (33.4%) received platinum-docetaxel/paclitaxel, whereas 300 (42.3%) received platinum-pemetrexed treatment. Furthermore, 219 (30.8%) and 257 (36.2%) of the patients received palliative radiotherapy and tyrosine-kinase inhibitor (TKI) treatment, respectively. The associations of these demographic characteristics and the known risk factors with NSCLC survival were also described in a previous publication^24^. The characteristics of demographic and clinical variables are described in Supplemental Table S2.

### NER rSNPs and NSCLC survival

The details of the eight (after excluding *DDB1/XPE* that does not have any rSNPs) core NER genes. We selected 25 rSNPs that are located in a regulatory region in either of the eight genes, and those rSNPs under investigation are shown in Table 1. We then performed the genotyping with DNA samples extracted from the whole blood cells. Call rates of the majority of the SNPs were >95%, except for three rSNPs (rs2607735, rs1007616 and rs7507745), which were then excluded from further analysis. In the univariate analysis without and multivariate analysis with adjustment for clinical variables, three rSNPs (*ERCC1* rs3212924, *XPC* rs2229090 and *ERCC4* rs1799798) consistently showed a significant association with either PFS or OS in NSCLC patients (Tables 2). Further subgroups analysis was performed for adenocarcinoma and squamous cell carcinoma patients, as well as by the dominant chemotherapy treatment, for these two histological types.

**Table 1:** Characteristics and functional prediction of the core NER genes and their regulatory region SNPs.

Gene	SNP ID	Chr.	Position	Gene location	Alleles^a^	TFBS^b^	Splicing^c^	miRNA	MAF^d^	Detected MAF	eQTL^e^	Call rate	HWE	Tagging SNPs
*ERCC1*	rs3212986	19	45409478	3′UTR	C > A	—	—	—	0.31	0.34	Y	99.9%	0.350	4
	rs2298881	19	45423658	Intron	C > A	Y	—	—	0.43	0.3	Y	93.0%	0.028	0
	rs3212924	19	45425445	5′ near gene	A > G	Y	—	—	0.28	0.31	Y	99.7%	0.868	0
	rs3212930	19	45424352	5′UTR	A > G	Y	—	—	0.12	0.1	Y	95.9%	0.894	0
*ERCC2/XPD*	rs50871	19	45359257	Intron	A > C	—	—	—	0.27	0.29	N	99.9%	0.081	0
	rs3916788	19	45371154	5′UTR	C > A	Y	—	—	0.51	0.48	Y	97.2%	0.159	0
	rs238416	19	45353791	Intron	C > T	—	—	—	0.47	0.47	Y	99.9%	0.010	4
	rs2097215	19	45372529	5′near gene	C > T	Y	—	—	0.49	0.5	Y	99.9%	0.066	12
*ERCC3/XPB*	rs4150477	2	127274970	Intron	A > G	—	—	—	0.45	0.41	Y	99.9%	0.001	26
	rs13385611	2	127247114	5′ near gene	T > C	Y	—	—	0.12	0.12	N	99.7%	0.437	17
	rs3738948	2	127260487	Intron	A > G	—	—	—	0.33	0.29	Y	99.6%	0.094	19
*ERCC4/XPF*	rs1799798	16	13920421	Intron	G > A	Y	—	—	0.15	0.12	N	100.0%	0.307	0
	rs3136038	16	13919522	5′ near gene	C > T	Y	—	—	0.22	0.23	Y	99.9%	0.317	42
*ERCC5/XPG*	rs751402	13	102845848	5′UTR	G > A	Y	Y	—	0.35	0.36	Y	99.9%	0.233	11
	rs2094258	13	102844409	Intron	C > T	Y	—	—	0.36	0.37	N	99.9%	0.151	9
	rs3759497	13	102844227	Intron	G > A	Y	—	—	0.37	0.4	Y	99.9%	0.043	0
	rs2296147	13	102846025	5′UTR	T > C	Y	—	—	0.23	0.21	Y	99.9%	0.981	10
	rs873601	13	102875987	3′UTR	A > G	—	Y	Y	0.48	0.5	N	100.0%	0.822	5
*XPC*	rs1982546	3	14175789	Intron	C > A	Y	—	—	0.25	0.26	N	99.6%	0.680	17
	rs2229090	3	14145845	3′UTR	G > C	—	—	Y	0.33	0.36	N	99.9%	0.797	6
	rs2607772	3	14177502	Intron	G > A	Y	—	—	0.38	0.37	N	99.9%	0.018	12
	rs2607775	3	14178595	5′UTR	C > G	Y	Y	—	0.05	0.04	Y	98.2%	0.297	19
*XPA*	rs1800975	9	97697296	5′UTR	T > C	Y	Y	—	0.48	0.46	Y	100.0%	0.963	13
	rs3176623	9	97698702	5′ near gene	C > A	Y	—	—	0.10	0.14	N	99.7%	0.178	0
*RAD23B*	rs7041137	9	107282291	Upstream	C > T	Y	—	—	0.17	0.22	N	99.9%	0.785	48
*DDB1/XPE*	none													

^a^Major > Minor allele; ^b^Transcription factor binding site; ^c^Enhance or abolish domain; ^d^Minor allele frequency in Chinese Han Beijing (CHB); ^e^Expression quantitative trait loci

Abbreviations: SNP, single nucleotide polymorphism; Chr, chromosome; MAF, minor allele frequency; eQTL, expression quantitative trait loci; HWE, Hardy-Weinberg equilibrium; UTR, untranslated region; NER, nucleotide excision repair.

**Table 2:** Association of NER rSNPs *ERCC1* rs3212924 A > G, *XPC* rs2229090 G > C and *ERCC4* rs1799798 G > A with progression free survival (PFS) and overall survival (OS) in Chinese NSCLC patients.

Gene	SNP	PFS					**OS**				
		Event/No.	MST (mo)	*P* ^a^	Adjusted HR^b^ (95% CI)	***P*** ^***b***^	**Event/No.**	**MST (mo)**	***P*** ^**a**^	**Adjusted HR** ^**c**^ **(95% CI)**	***P*** ^***c***^
***ERCC1***	**rs3212924**										
	AA	288/342	7.6	0.081	1.00 (ref.)		167/342	29.6	0.847	1.00 (ref.)	
	AG	267/305	6.3		**1.23 (1.04–1.46)**	**0.018**	139/305	27.0		1.13 (0.90–1.42)	0.307
	GG	47/61	7.6		1.10 (0.81–1.51)	0.537	25/61	25.5		0.95 (0.62–1.46)	0.826
	AG/GG	314/366	6.5	**0.030**	**1.21 (1.03–1.43)**	**0.021**		26.8	0.662	1.10 (0.88–1.36)	0.415
***XPC***	**rs2229090**										
	GG	257/293	6.5	0.101	1.00 (ref.)		138/293	29.8	0.459	1.00 (ref.)	
	GC	280/328	7.2		**0.79 (0.67–0.94)**	**0.009**	157/328	25.5		0.98 (0.77–1.23)	0.844
	CC	66/88	8.9		0.81 (0.61–1.07)	0.131	37/88	29.3		0.84 (0.58–1.22)	0.352
	GC/CC	346/416	7.4	0.063	**0.80 (0.68–0.94)**	**0.007**	194/416	25.9	0.922	0.94 (0.75–1.18)	0.586
***ERCC4***	**rs1799798**										
	GG	461/547	6.9	0.768	1.00 (ref.)		240/547	29.3	**0.028**	1.00 (ref.)	
	GA	132/149	7.5		0.93 (0.76–1.13)	0.475	88/149	21.6		**1.36 (1.07–1.75)**	**0.014**
	AA	11/14	8.5		0.79 (0.43–1.45)	0.441	4/14	28.8		0.77 (0.28–2.08)	0.604
	GA/AA	143/163	7.6	0.657	0.92 (0.76–1.11)	0.382	92/163	24.0	**0.032**	**1.32 (1.04–1.69)**	**0.026**
	Number of risk genotypes (NRGs)										
	0	167/205	8.1	**0.018**	1.00 (ref.)		97/205	28.2	0.625	1.00 (ref.)	
	1	299/347	7.0		**1.25 (1.03–1.51)**	**0.026**	166/347	28.1		1.24 (0.96–1.60)	0.365
	2	136/156	6.1		**1.50 (1.19–1.90)**	**0.0006**	68/156	28.6		1.14 (0.83–1.56)	0.431
	0 variant (LRi)	169/207	7.9	**0.023**	1.00 (ref.)		98/207	28.2	0.468	1.00 (ref.)	
	1-2 variants (HRi)	435/503	6.7		**1.32 (1.10–1.58)**	**0.003**	234/503	28.1		1.21 (0.95–1.54)	0.131

^a^ *P* value from Log-rank tests; ^b^Data were calculated using Cox hazards regression analysis, with a log-rank test adjusted for age-at-treatment, sex, smoking status, TNM stage, histological type, histologic grade, ECOG performance status, chemotherapy regimens, grade 3/4 chemotherapy toxicity and palliative radiotherapy; ^c^Data were calculated using Cox regression with adjustment for age at treatment, sex, TNM stage, smoking status, histological type, histologic grade, ECOG performance status, chemotherapy regimens, grade 3/4 chemotherapy toxicity, palliative radiotherapy and tyrosine-kinase inhibitor treatment. *P* < 0.05 are indicated in bold.

**Figure 1:**
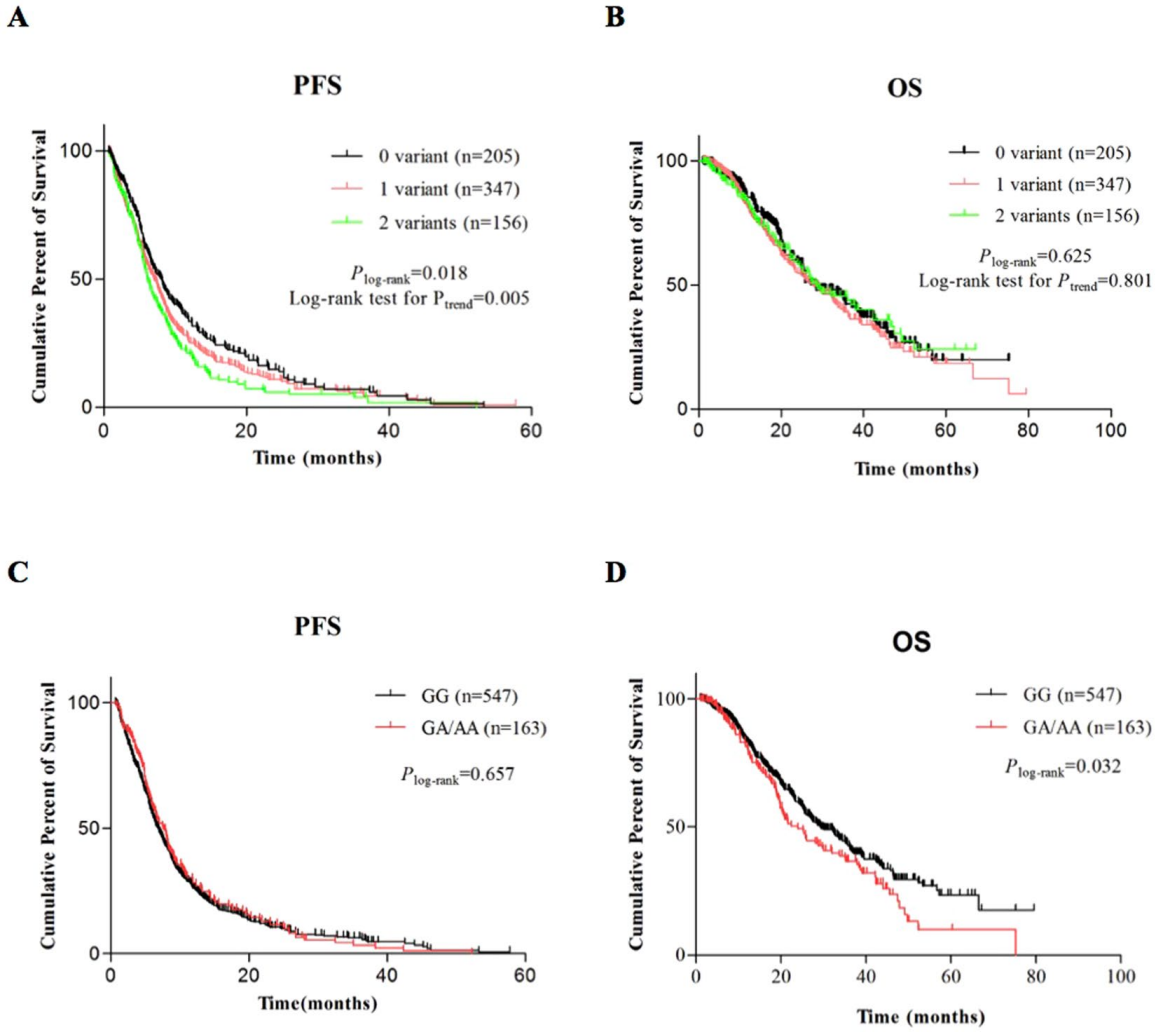
Kaplan-Meier survival curves for non-small cell lung cancer (NSCLC) patients by risk genotypes. (**A**) Progression-free survival (PFS) curves for a score of 0, 1, 2 for the number of risk genotypes (NRGs, i.e. rs3212924 AG/GG, rs2229090 GG); (**B**) Overall survival (OS) curve for a score of 0, 1, 2 for the NRGs (i.e. rs3212924 AG/GG, rs2229090 GG); (**C**) PFS curves for *ERCC4* rs1799798 risk genotypes (GA/AA vs. GG); (**D**) OS curves for *ERCC4* rs1799798 risk genotypes (GA/AA vs. GG).

Specifically, the *ERCC1* rs3212924 G allele was found to be significantly associated with a poor PFS [AG/GG vs. AA: median survival time (MST) 6.5 vs. 7.6 months, *P*_log-rank_ = 0.030; adjusted hazards ratio (HR_adj_) = 1.21, 95% CI = 1.03–1.43, *P*_adj_ = 0.021, under a dominant model] (Table 2). This variant was not significantly associated with PFS in patients with adenocarcinoma, nor in patients with squamous cell carcinoma alone in our dataset (Supplemental Table S3). The *XPC* rs2229090 C allele was associated with a longer PFS for all patients (GC/CC vs. GG: MST 7.4 vs. 6.5 months, *P*_log-rank_ = 0.063; HR_adj_ = 0.80, 95% CI = 0.68–0.94, *P*_adj_ = 0.007, under a dominant model) (Table 2). In the stratified analysis by histological type and treatment, this variant was significantly associated with a longer PFS only in adenocarcinoma patients alone and squamous cell carcinoma patients who had received docetaxel-cisplatin (GC/CC vs. GG: MST 7.4 vs.6.1 and 10.3 vs. 6.5, *P*_log-rank_ = 0.109 and 0.030, HR_adj_ = 0.79 and 0.44, 95% CI = 0.65–0.96 and 0.22–0.90, *P*_adj_ = 0.021 and 0.025, respectively) (Supplemental Tables S3 and S4). Patients carrying *ERCC4* rs1799798 GA/AA genotypes showed a significantly increased risk of death, compared with those with the GG genotype (MST 24 vs. 29.3 months, *P*_log-rank_ = 0.032; HR_adj_ = 1.32, 95% CI = 1.04–1.69, *P*_adj_ = 0.026, also under a dominant model) (Table 2; Fig. 1D). This variant has a borderline association with OS in adenocarcinoma patients, but not in squamous cell lung cancer patients, which is likely due to sample size reduction in the subgroup analysis (Supplemental Table S3). When we combined all risk genotypes into the number of risk genotypes (NRGs, i.e., the number of *ERCC1* rs3212924 GG/AG and *XPC* rs2229090 GG genotypes) for assessing their joint effect on PFS, the frequencies of patients with a score of 0, 1 or 2 for NRGs were 205, 347 and 156, respectively (Table 2). A dose-dependent trend was observed for patients carrying at least one of these genotypes and patients carrying two of these genotype had the highest risk for disease progression, compared with those carrying zero risk genotypes (HR_adj_ = 1.50, 95% CI = 1.19–1.90, *P*_log-rank_ = 0.017, *P*_adj_ = 0.0006) (Table 2; Fig. 1A). After dichotomizing patients into a low-risk (0 risk genotype) (LRi) or a high-risk (1–2 risk genotypes) (HRi) group, patients in the HRi group exhibited a significant shorter survival time before progression (HR_adj_ = 1.32, 95% CI = 1.10–1.58, *P*_adj_ = 0.003), compared to those in the LRi group (Table 2).

### Stratified analysis between the risk genotypes and NSCLC survival

Stratified analysis was also performed to assess differential effects of demographic or clinical variables (such as tumour histological type and treatment) on survival risk associated with genotype groups (LRi or HRi) or risk genotypes. Overall, the risk genotype group carriers (*ERCC1* rs3212924 AG/GG and *XPC* rs2229090 GG) tended to have a significantly increased risk of disease progression in subgroups of younger (≤58 years old), males, current smokers, TNM stage III, no radiotherapy, ECOG status 2, poorly differentiated, platinum-docetaxel/paclitaxel recipients. Most homogeneity tests did not provide any evidence to support for differences in HRs between the strata, except for the performance status (*P* = 0.006), which may be caused by unbalanced distribution of risk genotype groups between different subgroups. For *ERCC4* rs1799798 GA/AA carriers, an increased risk of death was observed in older patients (>58 years), non-smokers or former smokers, well-moderately differentiated tumours, and recipients of carboplatin-based or TKI chemotherapies, compared with the GG carriers (Supplemental Table S5).

### Correlations between *ERCC1* and *XPC* risk genotypes and mRNA expression levels

**Figure 2:**
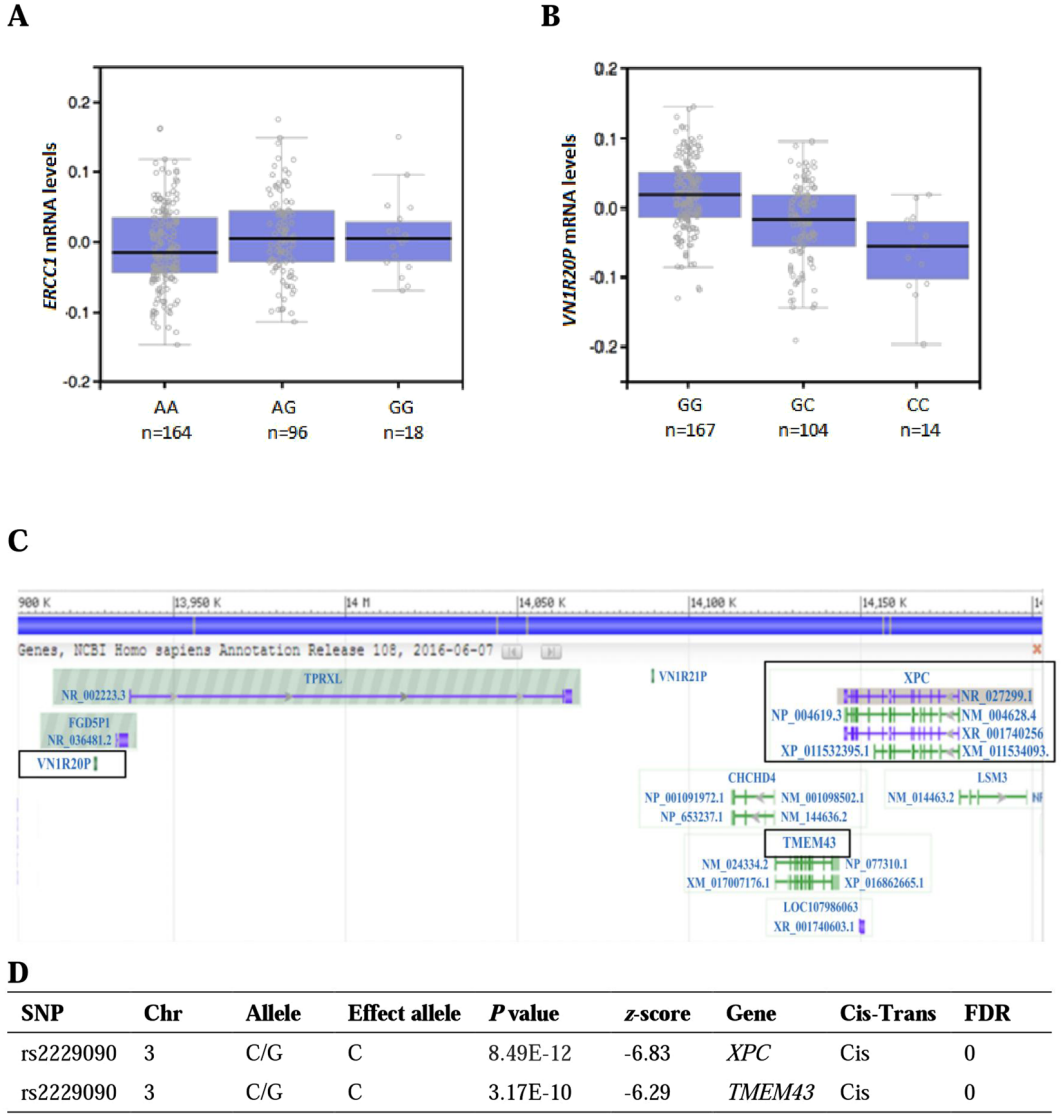
The eQTL analysis of *ERCC1* rs3212924 and *XPC* rs2229090 by using two publically available databases. (**A**) mRNA expression levels of *ERCC1* by rs3212924 genotypes in lung tissues from 278 individuals (P = 0.038, effect size = 0.13); (**B**) mRNA expression levels of *VN1R20P* by rs2229090 in tibial artery tissues from 285 individuals (P = 4.5E-12, effect size = −0.49); (**C**) Genomic position of *XPC*, *VN1R20P* and *TMEM43*; (**D**) *XPC* rs2229090 and gene expression in peripheral blood samples from 5,311 individuals and 2,775 replications (blood eQTL browser). Abbreviations: SNP, single nucleotide polymorphism; Chr, chromosome; FDR, false discovery rate.

To examine genotypic effect of the survival-associated rSNPs on gene expression, the eQTL analysis of the three NER rSNPs was further performed by using two publically available datasets. One included the GTEx samples of normal lung tissues, in which the *ERCC1* rs3212924 G allele was associated with a significantly higher *ERCC1* mRNA expression level (*P* = 0.038, effect size = 0.13) (Fig. 2A). The *XPC* rs2229090 protective C allele was associated with a lower expression level of *XPC* (*z*-score = −6.83, *P* = 8.39E-12) and a nearby gene *TMEM43* in peripheral blood cells (*z*-score = −6.29, *P* = 3.17E-10, Fig. 2D). Therefore, it is biologically plausible that the associations between those variants and NSCLC survival may be explained by the difference in gene expression levels regulated by those variants. That is, an increased expression of *ERCC1* was associated with a poor survival, whereas a decreased expression of *XPC* was associated with a better survival, and these support the notion that DNA repair is a double-edged sword.

## Discussion

According to the American Society of Clinical Oncology and National Comprehensive Cancer Network (NCCN) guidelines, lung cancer patients with a performance status of 0 or 1 should be treated with a combination of a platinum drug (cisplatin or carboplatin) and a non-platinum drug (e.g. paclitaxel) in the first-line therapy^25^. Cytotoxicity of platinum compounds results from formation of Plt-DNA adducts, leading to bulky distortion of DNA, destabilization of the double helix, inhibition of DNA replication, transcription and ultimately death of tumour cells^26^. Better clinical outcome was observed in patients with higher levels of Plt-DNA adducts in the tumours^13^. DNA repair capacity, particularly of the NER pathway, has been associated with the PBC efficacy. This is because NER primarily repairs bulky DNA adducts caused by mutagens and guanine-cisplatinium adducts formed during PBC^17^. Likewise, *in vitro* studies have also shown that NER is the major DNA repair pathway responsible for the repair of cisplatin-DNA damage^10^.

**Figure 3:**
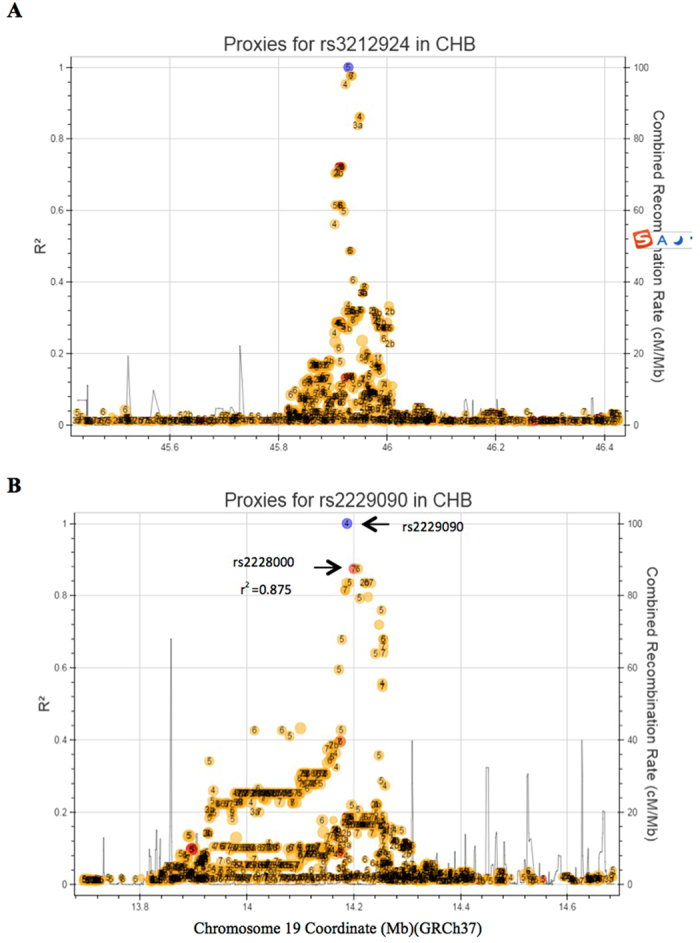
LD block of *ERCC1* and *XPC* risk variants. (**A**) LD block of *ERCC1* rs3212924; (**B**) LD block of *XPC* rs2229090. The blue circle indicates the SNP under study. R2 indicates the linkage disequilibrium value between the two pointed SNPs. Abbreviations: NER, nucleotide excision repair; LD, linkage disequilibrium.

Previous association studies on SNPs of NER genes and the survival of NSCLC have mainly focused on missense variants or coding regions of individual genes, with very few studies focusing on all the core genes in the pathway, linkage disequilibrium (LD) blocks or non-coding variants^27^. We adopted a hypothesis-based approach with a main focus on regulatory variants predicted to be biologically functional in NER. In the present study, we found that two rSNPs (*ERCC1* rs3212924 and *XPC* rs2229090) were associated with PFS and one rSNP (*ERCC4* rs1799798) associated with OS of NSCLC patients, and these associations were not previously reported for lung cancer. The rs3212924 variant resides at the upstream or an intron of different *ERCC1* transcripts, with a predicted function of altering transcription factor binding, which may further affect gene expression. Additional evidence from the eQTL analysis also indicated a significantly higher mRNA expression level in lung tissues containing the risk *ERCC1* G allele. Difference in gene expression by the rs3212924 G allele has been observed not only in lung tissue, but also in artery, skin, and ovary tissues, suggesting a genetically determined regulatory role of this variant in its gene expression. In the rs3212924 LD block, none of the other SNPs in high LD (r^2^ > 0.8) have been previously reported to be associated with cancer survival (Fig. 3A). Taken together, the associations between this variant with high tumour tissue levels of *ERCC1* mRNA may have led to cisplatin resistance^28, 29^, which may have independently affected disease progression in NSCLC patients.

The rs2229090 variant is located at the 3′UTR of *XPC*, and the G to C allele substitution is predicted to affect miRNA binding. In fact, the eQTL analysis indicated a genotypic effect of rs2229090 on expression of a pseudogene (Vomeronasal 1 Receptor 20 Pseudogene, *VN1R20P*) downstream of *XPC* in tibial artery tissues (Fig. 2B) and *XPC* expression in peripheral blood cells (Fig. 2D). In the corresponding LD block (Fig. 3B **)**, only variant rs2228000 (Ala499Val) (r^2^ = 0.875) was reported to be associated with survival of patients with various cancers including lung cancer^30–33^. Prior evidence indicated that subjects carrying rs2228000 CT/TT genotypes exhibited a better DNA repair capacity, and a poorer survival or risk of recurrence in oropharynx squamous cell carcinoma^34^ and acute myeloid leukaemia^35^. However, no detailed molecular mechanism of how rs2228000 T allele functions in these associations has ever been reported. It is likely that the phenotypic change of *XPC* function associated with rs2228000 may have been responsible for the observed association with rs2229090 that is within the same LD block (Fig. 3B). Because *XPC* plays a key role in recognizing DNA damage and initiation of the NER process, these collective findings suggest that *XPC* variants at the rs2229090 block may have an impact on PFS in NSCLC patients treated with PBC by affecting PBC outcome through changing *XPC* expression and thus the DNA repair capacity. These findings call for further functional studies to reveal the biological mechanisms underlying those associations.

In the subgroup analysis by histological type and chemotherapy treatment, *XPC* rs2229090 GC/CC exhibited a significant association with a longer PFS, while *ERCC4* rs1799798 GA genotype was significantly associated with a shorter OS in 477 adenocarcinoma patients, but not in 138 squamous cell carcinoma patients, suggesting a potential histological difference in genetic regulation of lung cancer survival outcome in response to the treatments. Although *XPC* rs2229090 GC/CC genotypes were significantly associated with PFS in squamous cell carcinoma patients who received docetaxel-cisplatin, the sample size of this treatment group was relatively small (n = 56); hence, this result needs to be interpreted with caution (Supplemental Table S4). It is also possible that the sample size in most of the subgroups was not large enough to reveal the real associations, suggesting that future larger validation studies are required to substantiate our findings.

The ROC curve prediction model for PFS incorporating *ERCC1* and *XPC* risk genotypes exhibited a statistically significant improvement in discriminatory power, compared with that of the clinical factors only (I/C AUC 0.59 vs. 0.58, *P* = 0.019) (Supplemental Fig. S1A and B; Table S7). There was a trend towards a higher AUC of ROC and C index in the genotype-inclusive prediction models for the five-year overall survival (Supplemental Fig. S1C and D; Table S7).

There are inherent limitations in the present study. First, the recruitment of patients treated in the same hospital may lead to selection bias in generalization of the results to the general population; therefore, additional results of patients from other hospitals of other populations are necessary to confirm our findings. Second, with the aim of studying potentially functional SNPs in the regulatory regions of eight NER core genes, we did not incorporate the other known effect of non-synonymous SNPs on survival outcome of NSCLC patients, although they are not in the LD block with the ones under investigation in the present study (except for rs2228080). Third, multiple testing correction was not conducted in the present study, because this was an exploratory study with a limited study power. Prospective studies in larger populations are warranted to substantiate the findings in the present study.

## Conclusions

The present study provided evidence that rSNPs in the core NER genes may modulate PBC-related survival outcome in Chinese NSCLC patients with an advanced stage disease. Potential gene regulation by rSNPs of two NER genes associated with outcomes of patients with NSCLC call for further functional studies to unravel the molecular mechanisms underlying the observed associations, which will also allow for further development of predictive biomarkers to facilitate personalized chemotherapy regime.

## Material and Methods

### Study populations

The present study was conducted on patients diagnosed with histologically advanced NSCLC from Fudan University Shanghai Cancer Centre (FUSCC) between February 1, 2009 and November 30, 2013. The recruitment criteria included the following: (1) unrelated Han Chinese with inoperable TNM stages III to IV tumours of NSCLC without prior history of cancer other than *in situ* carcinoma; (2) received PBC as the first-line treatment; (3) having Eastern Cooperative Oncology Group performance (ECOG) status 0 to 2 with laboratory testing for blood tests and uronoscopy in normal range; (4) no active infection and serious medical or psychological conditions that might prevent patients from adhering to treatment; and (5) patients with recent myocardial infarction, cardiac arrhythmia, active congestive heart failure or cerebral apoplexy, crankiness or depression were excluded from this study. The clinical data including age at treatment, sex, smoking history, ECOG performance, TNM stage, histological type and grade, chemotherapy regimens, radiotherapy, tyrosine-kinase (TKI) treatment were collected from patients’ medical records.

### Survival data

Survival data were collected from patients’ next of kin through a telephone follow-up and inpatient and outpatient clinical medical records. OS time was calculated from the starting date of the treatment until the date of the last follow-up or death. PFS time was measured from the starting date of the treatment until the last follow-up, progression of disease or death. Patients without progression were censored at the date of last contact. The median follow-up time was 32.1 months. The Institutional Review Board of FUSCC approved this study, with all methods performed in accordance with the guidelines and regulations of FUSCC. All participants provided an informed consent for using their blood samples in future research.

### Chemotherapy Regimens

All patients enrolled in the study were given the first-line PBC, which consists of ten combinations: cisplatin (75 mg/m^2^) or carboplatin (area under the curve [AUC] 6 mg/ml·min), administered with paclitaxel (175 mg/m^2^) on day 1 every 3 weeks, docetaxel (75 mg/m^2^) on day 1 every 3 weeks, gemcitabine (1250 mg/m^2^) on day 1 and 8 every 3 weeks, pemetrexed (500 mg/m^2^) on day 1 every 3 weeks or vinorelbine (25 mg/m^2^) on days 1 and 8 every 3 weeks, and cisplatin (100 mg/m^2^) or carboplatin (AUC 6 mg/ml·min) administered on day 1 every 4 weeks, in combination with etoposide (100 mg/m^2^) on days 1 to 3 every 4 weeks. All chemotherapeutic drugs were administered intravenously.

### SNP selection

To specifically explore the association between rSNPs in core NER genes and survival of NSCLC in response to PBC, all rSNPs were queried from the NER gene regions under the study by using SNP/GeneView in dbSNP database (http://www.ncbi.nlm.nih.gov/snp/) using the GRCh38 reference build of the human genome. A total of 25 rSNPs in eight (out of nine) core NER genes were chosen, with detailed characteristics of all investigated genes and rSNPs shown in (Table 1 and Supplemental Table S1 **)**. The selection criteria were based on the following: minor allele frequency (MAF) ≥ 5% in Han Chinese, in the regulatory region (5′ near gene, 5′UTR, intron, 3′ near gene, or 3′UTR), in low LD with each other (r^2^ < 0.8), have predicted functions (transcription factor binding site, splicing, miRNA binding site or significant eQTL) by SNPinfo (http://snpinfo.niehs.nih.gov/snpfunc.htm) and GTEx portal (http://www.gtexportal.org/home/). A full list of the NER genes analysed in this study, their region coordinates, their start sites and stop sites, and the characteristics of genotyped variants are summarized in Table 1.

### SNPseq genotypin

Genomic DNA was extracted from the whole blood of all study subjects by using DNA Blood Mini Kit (Qiagen, Valencia, CA). The purity [optical density (OD)_260/280_ at 1.7~2.0] and concentration ( >20 ng/μl) that met the sequencing requirements. Genotyping of all rSNPs was conducted by FastTarget, a next generation sequencing-based method using Illumina Miseq. 2000 Platform (2 × 250 bp, Illumina, CA, USA). Prior to sequencing, 5% of the samples were randomly selected and subjected to 1% agarose gel electrophoresis quality control. Genomic regions containing the investigating rSNPs were amplified using the FastTarget^TM^ technology (Genesky Biotechnologies Inc, Shanghai, China). A total of 25 amplicons were amplified, with the primers information attached in Supplemental Table S6. After multiple PCR reactions, DNA fragments were ligated with the adaptor by using Q5 DNA polymerase Kit (New England Biolabs, MA, USA), and further purified by Agencourt AMPure XP (Beckman Coulter, CA, USA). Next-generation sequencing of the amplification products was carried out by MiSeq 2000 Sequencer (Illumina, Inc., San Diego, CA, USA), following the manufacturer’s standard protocols. Sequencing depth of more than 30x was achieved for over 90% of the samples. Output sequence data were trimmed and then compared with fragment reference sequences (hg19) using the Blat program^28^. Burrows- Wheeler Aligner (BWA, V 0.7.5a) was used to map the reads^36^, followed by Sequence Alignment/Map (SAM)-to-BAM conversion, sorting, and removal of duplicates using SAM tools (v0.1.19)^37^. Combined rSNP calling was performed on the resulting BAM files using Genome Analysis Toolkit (GATK, https://software.broadinstitute.org/gatk/best-practices/) and VarScan programs^38^. Finally SNP annotation was done by the Annovar program^39^.

### Statistical analysis

The association between each genetic variant and PFS/OS was estimated by Cox proportional hazards regression model, calculated as HRs with their corresponding 95% CIs. The covariates used for adjusted HR for PFS included age-at-treatment, sex, smoking status, TNM stage, histological type, histologic grade, ECOG performance status, chemotherapy regimens, grade 3/4 chemotherapy toxicity and palliative radiotherapy, whereas TKI treatment was included for adjusted HR for OS in addition to the covariates mentioned above. Kaplan-Meier test was used to assess each genetic variant on the cumulative probability of PFS and OS^40^. Log-rank test was used to examine the difference in survival between groups. The observed associations were stratified by selected demographic and clinical variables. The heterogeneity between subgroups was assessed by the *χ* ^2^-based Q test. For survival prediction model construction, independent predictors including selected clinical variables and genetic variants were included. ROC analysis was used to compare sensitivity and specificity of the OS and PFS prediction by the included parameters. Predictive values of selected variables were evaluated by I/D AUC of the ROC curves for censored data and C index for comparison of survival models. The I/D ROC and I/D AUC were calculated and plotted by RisksetROC package of R software (version 3.2.3; The R Foundation for Statistical Computing)^41^. All statistical analyses were performed by SAS software (version 9.4; SAS Institute, Cary, NC). Unless stated otherwise, all *P* values were two-sided with a significance level of *P* < 0.05.

### The eQTL analysis

Two large-scale eQTL datasets were used to assess the correlation between survival-related genetic variants and NER gene expression levels: one is the GTEx project using 278 lung tissue samples, and the other is the blood eQTL browser (http://www.genenetwork.nl/bloodeqtlbrowser/) encompassing 5,311 individuals and 2,775 replicates^42^.

## Electronic supplementary material

Supplementary Information
