# Supplementary material for: Genetic variants in ERCC1 and XPC predict survival outcome of non-small cell lung cancer patients treated with platinum-based therapy
Source: Sci Rep. 2017 Sep 6;7:10702. doi: 10.1038/s41598-017-10800-5 (PMC5587538; doi:10.1038/s41598-017-10800-5)
Supplement: Supplementary file 1 — Supplementary Information [file 41598_2017_10800_MOESM1_ESM.doc]

**Genetic variants in *ERCC1* and *XPC* predict survival outcome of non-small cell lung cancer patients treated with platinum-based therapy**

Ruoxin Zhang^*^, Ming Jia^*^, Huijing Xue, Yuan Xu, Mengyun Wang, Meiling Zhu, Menghong Sun, Jianhua Chang^**^ and Qingyi Wei^**^

| **No.** | **Gene** | **Region coordinates** | **No. of total SNPs**  **analyzed** | **No. of validated SNPs**  **with predicted function** | **No. of rSNPs**  **Selected for genotyping** |
| --- | --- | --- | --- | --- | --- |
| 1 | *ERCC1* | 45406837-45425905 | 1608 | 36 | 4 |
| 2 | *ERCC2/XPD* | 45350896-45372529 | 2440 | 36 | 4 |
| 3 | *ERCC3/XPB* | 127256790-127296171 | 2525 | 44 | 3 |
| 4 | *ERCC4/XPF* | 13918164-13952831 | 2471 | 62 | 2 |
| 5 | *ERCC5/XPG* | 102843853-102876496 | 2472 | 63 | 5 |
| 6 | *XPC* | 14144657-14180634 | 2625 | 55 | 4 |
| 7 | *XPA* | 97674414-97699337 | 1501 | 67 | 2 |
| 8 | *RAD23B* | 107281269-107332686 | 3315 | 102 | 1 |
| 9 | *DDB1/XPE* | 61298995-61335208 | 2624 | 34 | 0 |
| **Supplemental Table S1. Characteristics of all the genes and rSNPs analyzed.**  Abbreviations: SNP, single nucleotide polymorphism; rSNP, regulatory SNP. | | | | | |

| **Patient characteristics** |  | **Progression free survival (PFS)** | | | |  | **Overall survival (OS)** | | | |  | |
| --- | --- | --- | --- | --- | --- | --- | --- | --- | --- | --- | --- | --- |
|  |  | **MST (mo)** | ***P^a^*** | **HR (95% CI)** | ***P^b^*** |  | **MST (mo)** | ***P^a^*** | **HR (95% CI)** | ***P^b^*** |  | |
| All subjects |  | 7 |  |  |  |  | 28.2 |  |  |  |  | |
| Median age range, yr | ≤58 | 6.6 | 0.105 | 1.00 (ref.) |  |  | 28.6 | 0.373 | 1.00 (ref.) |  |  | |
|  | >58 | 7.5 |  | 0.88 (0.75-1.03) | 0.107 |  | 26.8 |  | 1.10 (0.89-1.37) | 0.372 |  | |
| Sex | Male | 6.5 | **0.004** | 1.00 (ref.) |  |  | 25.3 | **0.0002** | 1.00 (ref.) |  |  | |
|  | Female | 8.4 |  | **0.77 (0.64-0.92)** | **0.004** |  | 33.7 |  | **0.62 (0.49-0.80)** | **0.0002** |  | |
| Performance status | 0-1 | 7.7 | **0.034** | 1.00 (ref.) |  |  | 29.1 | 0.125 | 1.00 (ref.) |  |  | |
|  | 2 | 6.5 |  | **1.19 (1.01-1.40)** | **0.035** |  | 26.3 |  | 1.19 (0.95-1.48) | 0.126 |  | |
| Smoking status^c^ | Nonsmokers | 8 | **0.015** | 1.00 (ref.) |  |  | 33.7 | **<0.0001** | 1.00 (ref.) |  |  | |
|  | Former-smokers | 7.3 |  | 1.18 (0.83-1.67) | 0.35 |  | 24.9 |  | 1.44 (0.89-2.33) | 0.134 |  | |
|  | Current-smokers | 6 |  | **1.27 (1.08-1.50)** | **0.004** |  | 22.8 |  | **1.65 (1.32-2.07)** | **<0.0001** |  | |
| TNM | III | 8.2 | **0.001** | 1.00 (ref.) |  |  | 36.2 | **0.005** | 1.00 (ref.) |  |  | |
|  | IV | 6.2 |  | **1.33 (1.13-1.57)** | **0.001** |  | 25.5 |  | **1.38 (1.10-1.72)** | **0.005** |  | |
| Histological type | Squamous cell carcinoma | 7.7 | 0.86 | 1.00 (ref.) |  |  | 25.8 | 0.172 | 1.00 (ref.) |  |  | |
|  | Adenocarcinoma | 6.9 |  | 1.05 (0.86-1.30) | 0.621 |  | 29.3 |  | 0.93 (0.71-1.24) | 0.633 |  | |
|  | Other^d^ | 6.3 |  | 1.14 (0.83-1.56) | 0.432 |  | 20.3 |  | 1.41 (0.92-2.15) | 0.112 |  | |
|  | NSCLC-NOS | 8.2 |  | 0.97 (0.60-1.56) | 0.902 |  | 33.7 |  | 1.07 (0.53-2.16) | 0.843 |  | |
| Histologic grade | Poorly differentiated | 6.8 | 0.97 | 1.00 (ref.) |  |  | 27.2 | 0.149 | 1.00 (ref.) |  |  | |
|  | Well-moderately differentiated | 7.4 |  | 1.00 (0.85-1.17) | 0.97 |  | 32.1 |  | 0.85 (0.68-1.06) | 0.149 |  | |
| Chemotherapy regimen 1 | Platinum-docetaxel/  paclitaxel | 6.9 | 0.575 | 1.00 (ref.) |  |  | 29.8 | 0.166 | 1.00 (ref.) |  |  | |
|  | Platinum-etoposide | 6.3 |  | 1.25 (0.52-3.05) | 0.618 |  | NA |  | 1.13 (0.16-8.14) | 0.904 |  | |
|  | Platinum-gemcitabine | 6.6 |  | 0.97 (0.78-1.22) | 0.813 |  | 22.2 |  | **1.46 (1.08-1.96)** | **0.014** |  | |
|  | Platinum-vinorelbine | 9.5 |  | 0.82 (0.56-1.20) | 0.299 |  | 28.8 |  | 0.99 (0.56-1.74) | 0.97 |  | |
|  | Platinum-pemetrexed | 7 |  | 0.88 (0.73-1.06) | 0.19 |  | 28.6 |  | 1.16 (0.90-1.49) | 0.264 |  | |
| Chemotherapy regimen 2 | Cisplatin-  combinations | 7 | 0.179 | 1.00 (ref.) |  |  | 28.6 | 0.585 | 1.00 (ref.) |  |  | |
|  | Carboplatin-  combinations | 6.9 |  | 1.15 (0.94-1.40) | 0.18 |  | 26.3 |  | 0.93 (0.70-1.22) | 0.586 |  | |
| Grade 3/4 chemotherapy toxicity | No | 7.5 | 0.358 | 1.00 (ref.) |  |  | 28.6 | 0.92 | 1.00 (ref.) |  |  | |
|  | Yes | 6.5 |  | 1.08 (0.92-1.28) | 0.359 |  | 27.6 |  | 1.01 (0.81-1.27) | 0.92 |  | |
| Palliative radiotherapy | No | 6.1 | **<0.0001** | 1.00 (ref.) |  |  | 25.2 | **0.0002** | 1.00 (ref.) |  |  | |
|  | Yes | 8.8 |  | 0.67 (0.56-0.80) | **<0.0001** |  | 36.1 |  | **0.63 (0.49-0.81)** | **0.0003** |  | |
| TKI treatment | No |  |  |  |  |  | 25.5 | **0.024** | 1.00 (ref.) |  |  | |
|  | Yes |  |  |  |  |  | 33.7 |  | **0.77 (0.62-0.97)** | **0.024** |  | |
| **Supplemental Table S2. Association between clinical characteristics and PFS and OS of patients with advanced NSCLC.** ^a^ *P* Value for Log-rank tests. ^b^ *P* Value for univariate Cox hazards regression analysis. ^c^ Patients were categorized as never smokers (< 100 lifetime cigarettes), former smokers (quit ≥ 1 year ago), or current smokers (quit < 1 year ago) at the time of diagnosis. ^d^ Other carcinomas include adenosquamocarcinoma, mixed cell, neuroendocrine carcinoma, and undifferentiated carcinoma. Values shown in **bold** if *P*< 0.05.  Abbreviations: PFS, progression free survival; OS, overall survival; NSCLC, non-small cell lung cancer; HR, hazards ratio; CI, confidence interval; MST, median survival time; mo, month; yr, year; NSCLC-NOS, non-small cell lung cancer not otherwise specified; TKI, tyrosine kinase inhibitor. | | | | | | | | | | | |  |

| **Gene** | **SNP** | | **PFS** | | | | | |  | **OS** | | | | | | | | | | | | | | | | | |  | | |
| --- | --- | --- | --- | --- | --- | --- | --- | --- | --- | --- | --- | --- | --- | --- | --- | --- | --- | --- | --- | --- | --- | --- | --- | --- | --- | --- | --- | --- | --- | --- |
|  |  | | **Event/No** | **MST (mo)** | ***P*^a^** | **Adjusted HR^b^ (95% CI)** | ***P^b^*** | |  | **Event/No** | | | | | **MST (mo)** | | | ***P*^a^** | | | **Adjusted HR^c^**  **(95% CI)** | | | | | ***P^c^*** | |  | | |
| ***ERCC1*** | **rs3212924** | | |  |  |  |  | |  |  | | | | |  | | |  | | |  | | | | |  | |  | | |
|  | Adenocarcinoma (476) | | | | | | | | | | | | | | | | | | | | | | | | | |  | | | |
|  | AA | | 199/233 | 7.2 | 0.256 | 1.00 (ref.) |  | |  | 115/233 | | | | | 30.1 | | | 0.977 | | | 1.00 (ref.) | | | |  | | |  | | |
|  | AG | | 182/204 | 6.2 |  | 1.16 (0.94-1.42) | 0.163 | |  | 96/204 | | | | | 28.1 | | |  | | | 1.09 (0.83-1.43) | | | | 0.550 | | |  | | |
|  | GG | | 31/39 | 8.5 |  | 0.98 (0.66-1.45) | 0.921 | |  | 16/39 | | | | | 25.6 | | |  | | | 0.97 (0.57-1.64) | | | | 0.903 | | |  | | |
|  | AG/GG | | 213/243 | 6.7 | 0.132 | 1.13 (0.93-1.38) | 0.211 | |  | 112/243 | | | | | 28.1 | | | 0.911 | | | 1.07 (0.82-1.39) | | | | 0.636 | | |  | | |
|  | Squamous cell carcinoma (138) | | | | | | | | | | | | | | | | | | | | | | | | | |  | | | |
|  | AA | | 55/65 | 7.7 | 0.954 | 1.00 (ref.) |  | |  | 30/65 | | | | | 25.8 | | | 0.791 | | | 1.00 (ref.) | | | |  | | |  | | |
|  | AG | | 48/59 | 8.3 |  | 1.11 (0.68-1.83) | 0.677 | |  | 27/59 | | | | | 25.8 | | |  | | | 1.18 (0.63-2.21) | | | | 0.604 | | |  | | |
|  | GG | | 10/14 | 7.5 |  | 1.01 (0.49-2.07) | 0.983 | |  | 5/14 | | | | | 49.1 | | |  | | | 1.18 (0.42-3.30) | | | | 0.754 | | |  | | |
|  | AG/GG | | 58/73 | 7.5 | 0.861 | 1.08 (0.69-1.70) | 0.734 | |  | 32/73 | | | | | 25.8 | | | 0.763 | | | 1.18 (0.66-2.12) | | | | 0.580 | | |  | | |
| ***XPC*** | **rs2229090** | | |  |  |  |  | |  |  | | | | |  | | |  | | |  | | | |  | | |  | | |
|  | Adenocarcinoma (477) | | | | | | | | | | | | | | | | | | | | | | | | | |  | | | |
|  | GG | | 178/199 | 6.1 | 0.160 | 1.00 (ref.) |  | |  | 94/199 | | | | | 30.1 | | | 0.177 | | | 1.00 (ref.) | | | |  | | |  | | |
|  | GC | | 188/218 | 7.2 |  | **0.78 (0.63-0.97)** | **0.025** | |  | 109/218 | | | | | 25.5 | | |  | | | 1.10 (0.83-1.46) | | | | 0.522 | | |  | | |
|  | CC | | 47/60 | 8.9 |  | 0.81 (0.58-1.13) | 0.210 | |  | 25/60 | | | | | 44.0 | | |  | | | 0.70 (0.44-1.12) | | | | 0.134 | | |  | | |
|  | GC/CC | | 235/278 | 7.4 | 0.109 | **0.79 (0.65-0.96)** | **0.021** | |  | 134/278 | | | | | 26.3 | | | 0.526 | | | 0.99 (0.75-1.30) | | | | 0.949 | | |  | | |
|  | Squamous cell carcinoma (138) | | | | | | | | | | | | | | | | | | | | | | | | | |  | | | |
|  | GG | 46/54 | | 7.3 | 0.475 | 1.00 (ref.) |  |  | | | 23/54 | | | 25.4 | | | | | 0.869 | 1.00 (ref.) | | | |  | | | | |  | |
|  | GC | 56/69 | | 8.2 |  | 0.70 (046-1.07) | 0.098 |  | | | 31/69 | | | 25.8 | | | | |  | 0.98 (0.54-1.76) | | | | 0.934 | | | | |  | |
|  | CC | 11/15 | | 9.5 |  | 0.76 (0.37-1.56) | 0.459 |  | | | 8/15 | | | 36.2 | | | | |  | 1.19 (0.52-2.75) | | | | 0.681 | | | | |  | |
|  | GC/CC | 67/84 | | 8.2 | 0.248 | 0.71 (0.47-1.07) | 0.099 |  | | | 39/84 | | | 25.8 | | | | | 0.942 | 1.02 (0.59-1.78) | | | | 0.943 | | | | |  | |
| ***ERCC4*** | **rs1799798** | | |  |  |  |  |  | | |  | | |  | | | | |  |  | | | |  | | | | |  | |
|  | Adenocarcinoma (478) | | | | | | | | | | | | | | | | | | | | | | | | | | | | |  |
|  | GG | | 312/365 | 6.9 | 0.524 | 1.00 (ref.) |  | |  | | | | 165/365 | | | | 29.6 | | 0.144 | | | | 1.00 (ref.) | | | |  | |  | |
|  | GA | | 94/103 | 6.7 |  | 1.06 (0.84-1.35) | 0.622 | |  | | | | 60/103 | | | | 24 | |  | | | | **1.37 (1.01-1.85)** | | | | **0.042** | |  | |
|  | AA | | 8/10 | 8.7 |  | 0.73 (0.35-1.52) | 0.404 | |  | | | | 3/10 | | | | - | |  | | | | 0.85 (0.26-2.75) | | | | 0.786 | |  | |
|  | GA/AA | | 102/113 | 6.9 | 0.558 | 1.03 (0.82-1.30) | 0.814 | |  | | | | 63/113 | | | | 26 | | 0.117 | | | | 1.33 (0.99-1.80) | | | | 0.059 | |  | |
|  | Squamous cell carcinoma (138) | | | | | | | | | | | | | | | | | | | | | | | | | |  | | | |
|  | GG | | 89/112 | 7.3 | 0.661 | 1.00 (ref.) |  | |  | | | 45/112 | | | | 35.6 | | | 0.403 | | | 1.00 (ref.) | | | | |  | | | |
|  | GA | | 23/25 | 9.5 |  | 0.65 (0.38-1.11) | 0.112 | |  | | | 16/25 | | | | 17.5 | | |  | | | 1.38 (0.74-2.58) | | | | | 0.310 | | | |
|  | AA | | 1/1 | 7.8 |  | 1.39 (0.17-11.42) | 0.759 | |  | | | 1/1 | | | | 25.4 | | |  | | | 2.68 (0.30-24.05) | | | | | 0.380 | | | |
|  | GA/AA | | 24/26 | 9 | 0.435 | 0.67 (0.39-1.13) | 0.130 | |  | | | 17/26 | | | | 25.4 | | | 0.181 | | | 1.42 (0.77-2.62) | | | | | 0.261 | | | |

**Supplemental Table S3. Association of NER rSNPs *ERCC1* rs3212924 A>G, *XPC* rs2229090 G>C and *ERCC4* rs1799798 G>A with progression free survival (PFS) and overall survival (OS) in adenocarcinoma and squamous cell lung carcinoma patients.** ^a^ *P* Value for Log-rank tests. ^b^ Data were calculated using Cox hazards regression analysis, with a log-rank test adjusted for age-at-treatment, sex, smoking status, TNM stage, histologic grade, ECOG performance status, chemotherapy regimens, grade 3/4 chemotherapy toxicity and palliative radiotherapy; ^c^ Data were calculated using Cox regression with adjustment for age at treatment, sex, TNM stage, smoking status, histologic grade, ECOG performance status, chemotherapy regimens, grade 3/4 chemotherapy toxicity, palliative radiotherapy and tyrosine-kinase inhibitor treatment. Values shown in **bold** if *P*< 0.05.

Abbreviations: SNP, single nucleotide polymorphism; PFS, progression free survival; OS, overall survival; MST, median survival time; mo, month; CI,

confidence interval; HR, hazards ratio.

| **Gene** | **SNP** | **PFS** | | | | |  | | **OS** | | | | | |
| --- | --- | --- | --- | --- | --- | --- | --- | --- | --- | --- | --- | --- | --- | --- |
|  |  | **Event/No** | **MST (mo)** | ***P*^a^** | **Adjusted HR^b^ (95% CI)** | ***b^c^*** |  | | **Event/No** | | **MST (mo)** | ***P*^a^** | **Adjusted HR^c^ (95% CI)** | ***P^c^*** |
| ***ERCC1*** | **rs3212924** |  |  |  |  |  |  | |  | |  |  |  |  |
|  | Pemetrexed-cisplatin recipients in adenocarcinoma (243) | | | | |  |  | |  | |  |  |  |  |
|  | AA | 93/116 | 7.5 | 0.134 | 1.00 (ref.) |  |  | | 59/116 | | 30.1 | 0.924 | 1.00 (ref.) |  |
|  | AG | 97/109 | 6.1 |  | 1.27 (0.94-1.70) | 0.119 |  | | 54/109 | | 27 |  | 1.29 (0.88-1.89) | 0.200 |
|  | GG | 12/17 | 9.0 |  | 0.96 (0.52-1.78) | 0.891 |  | | 8/17 | | 25.5 |  | 1.36 (0.63-2.93) | 0.439 |
|  | AG/GG | 109/126 | 6.6 | 0.129 | 1.23 (0.92-1.64) | 0.156 |  | | 62/126 | | 27 | 0.693 | 1.31 (0.90-1.91) | 0.154 |
|  | Docetaxel-cisplatin recipients in squamous cell carcinoma (56) | | | | | | | | | | | | | |
|  | AA | 21/27 | 7.9 | 0.271 | 1.00 (ref.) |  |  | | 12/27 | | 35.6 | 0.624 | 1.00 (ref.) |  |
|  | AG | 20/23 | 6.7 |  | 1.43 （0.61-3.34） | 0.415 |  | | 9/23 | | 32.0 |  | 1.18 (0.41-3.45) | 0.760 |
|  | GG | 3/6 | 9.5 |  | 0.70 （0.16-3.02） | 0.636 |  | | 2/6 | | 49.1 |  | 0.90 (0.14-5.68) | 0.915 |
|  | AG/GG | 23/29 | 8.3 | 0.648 | 1.27 （0.55-2.94） | 0.584 |  | | 11/29 | | 36.2 | 0.883 | 1.12 (0.40-3.14) | 0.823 |
| ***XPC*** | **rs2229090** |  |  |  |  |  |  | |  | |  |  |  |  |
|  | Pemetrexed-cisplatin recipients in adenocarcinoma (243) | | | | | | | | | | | | | |
|  | GG | 83/92 | 6.3 | 0.367 | 1.00 (ref.) |  | |  | | 46/92 | 32.1 | 0.509 | 1.00 (ref.) |  |
|  | GC | 92/113 | 6.6 |  | 0.83 (0.61-1.13) | 0.238 | |  | | 57/113 | 25.5 |  | 0.99 (0.64-1.51) | 0.954 |
|  | CC | 28/38 | 8.9 |  | 0.72 (0.46-1.12) | 0.148 | |  | | 19/38 | 28.1 |  | 0.89 (0.51-1.57) | 0.693 |
|  | GC/CC | 120/151 | 7.4 | 0.450 | 0.80 (0.60-1.07) | 0.136 | |  | | 76/151 | 25.5 | 0.275 | 0.96 (0.64-1.44) | 0.845 |
|  | Docetaxel-cisplatin recipients in squamous cell carcinoma (56) | | | | | | | | | | | | | |
|  | GG | 16/20 | 6.5 | 0.073 | 1.00 (ref.) |  | |  | | 8/20 | 49.1 | 0.898 | 1.00 (ref.) |  |
|  | GC | 23/28 | 10.3 |  | **0.40 (0.19-0.84)** | **0.016** | |  | | 12/28 | 35.6 |  | 0.54 (0.19-1.57) | 0.257 |
|  | CC | 5/8 | 6.0 |  | 0.83 (0.25-2.77) | 0.760 | |  | | 3/8 | 39.6 |  | 0.61 (0.13-2.77) | 0.522 |
|  | GC/CC | 28/36 | 10.3 | **0.030** | **0.44 (0.22-0.90)** | **0.025** | |  | | 15/36 | 36.2 | 0.643 | 0.56 (0.21-1.47) | 0.237 |
| ***ERCC4*** | **rs1799798** |  |  |  |  |  | |  | |  |  |  |  |  |
|  | Pemetrexed-cisplatin recipients in adenocarcinoma (243) | | | | | | | | | | | | | |
|  | GG | 153/186 | 7.4 | 0.462 | 1.00 (ref.) |  | |  | | 91/186 | 28.6 | 0.457 | 1.00 (ref.) |  |
|  | GA | 46/53 | 6.2 |  | 1.13 (0.81-1.60) | 0.470 | |  | | 30/53 | 22.4 |  | 1.26 (0.82-1.93) | 0.290 |
|  | AA | 4/4 | 8.0 |  | 1.81 (0.64-5.10) | 0.262 | |  | | 1/4 | - |  | 0.93 (0.13-6.88) | 0.941 |
|  | GA/AA | 50/57 | 6.6 | 0.243 | 1.17 (0.84-1.63) | 0.358 | |  | | 31/57 | 24.0 | 0.547 | 1.25 (0.82-1.90) | 0.308 |
|  | Docetaxel-cisplatin recipients in squamous cell carcinoma (56) | | | | | | | | | | | | | |
|  | GG | 33/43 | 7.5 | 0.988 | 1.00 (ref.) |  | |  | | 16/43 | 39.0 | 0.272 | 1.00 (ref.) |  |
|  | GA | 11/13 | 10.4 |  | 0.51 （0.19-1.36） | 0.177 | |  | | 7/13 | 16.3 |  | 1.36 (0.51-3.66) | 0.539 |
|  | AA | - |  |  |  |  | |  | |  | - |  |  |  |
|  | GA/AA | 11/13 | 10.4 | 0.988 | 0.51 （0.19-1.36） | 0.177 | |  | | 7/13 | 16.3 | 0.272 | 1.36 (0.51-3.66) | 0.539 |

**Supplemental Table S4. Association of NER rSNPs *ERCC1* rs3212924 A>G, *XPC* rs2229090 G>C and *ERCC4* rs1799798 G>A with progression free survival (PFS) and overall survival (OS) in adenocarcinoma patients receiving** **pemetrexed-cisplatin and squamous cell carcinoma patients receiving docetaxel-cisplatin.** ^a^ *P* Value for Log-rank tests. ^b^ Data were calculated using Cox hazards regression analysis, with a log-rank test adjusted for age-at-treatment, sex, smoking status, TNM stage, histologic grade, ECOG performance status, grade 3/4 chemotherapy toxicity and palliative radiotherapy; ^c^ Data were calculated using Cox regression with adjustment for age at treatment, sex, TNM stage, smoking status, histologic grade, ECOG performance status,, grade 3/4 chemotherapy toxicity, palliative radiotherapy and tyrosine-kinase inhibitor treatment. Values shown in **bold** if *P*< 0.05.

Abbreviations: SNP, single nucleotide polymorphism; PFS, progression free survival; OS, overall survival; MST, median survival time; mo, month; CI,

confidence interval; HR, hazards ratio.

| **Variables** | | **rs3212924 and rs2229090 LRi/HRi and PFS** | | | | |  | **rs1799798 GA/AA vs. GG and OS** | | | | |
| --- | --- | --- | --- | --- | --- | --- | --- | --- | --- | --- | --- | --- |
|  |  | **No. of**  **patients (LRi/HRi)** | **Progression no. (LRi/HRi)** | **HR^a^**  **(95% CI)** | ***P*^a^** | ***P*_hom_** |  | **No. of patients** | **Progression no. (GA/AA vs. GG)** | **HR^b^(95% CI)** | ***P*^b^** | ***P*_hom_** |
| Age | ≤58 | 110/275 | 93/238 | **1.34 (1.03-1.74)** | **0.029** |  |  | 290/95 | 126/46 | 1.10 (0.78-1.56) | 0.587 |  |
|  | >58 | 97/228 | 76/197 | 1.19 (0.90-1.560) | 0.223 | 0.532 |  | 257/68 | 114/46 | **1.66 (1.15-2.39)** | **0.006** | 0.112 |
| Sex | Male | 148/360 | 123/309 | **1.34 (1.08-1.67)** | **0.008** |  |  | 398/110 | 180/67 | 1.29 (0.96-1.72) | 0.087 |  |
|  | Female | 59/143 | 46/126 | 1.28 (0.90-1.83) | 0.177 | 0.822 |  | 149/53 | 60/25 | 1.62 (0.97-2.71) | 0.064 | 0.441 |
| Performance status | 0-1 | 113/294 | 92/254 | 1.02 (0.80-1.31) | 0.87 |  |  | 314/93 | 142/50 | 1.24 (0.88-1.74) | 0.213 |  |
|  | 2 | 94/209 | 77/181 | **1.76 (1.31-2.36)** | **<0.001** | 0.006 |  | 233/42 | 98/42 | 1.27 (0.86-1.87) | 0.231 | 0.932 |
| Smoking status^c^ | Nonsmokers | 91/243 | 69/212 | 1.28 (0.960-1.70) | 0.094 |  |  | 250/84 | 102/40 | **1.48 (1.01-2.19)** | **0.047** |  |
|  | Former-smokers | 14/27 | 11/25 | 2.06 (0.74-5.78) | 0.169 |  |  | 36/5 | 15/4 | **12.84 (1.58-104.25)** | **0.017** |  |
|  | Current-smokers | 102/233 | 89/198 | **1.36 (1.04-1.77)** | **0.024** | 0.674 |  | 261/74 | 123/48 | 1.14 (0.81-1.61) | 0.459 | 0.062 |
| TNM | III | 81/207 | 63/171 | 1.46 (1.06-2.01) | **0.020** |  |  | 222/66 | 84/38 | 1.47 (0.97-2.23) | 0.072 |  |
|  | IV | 126/296 | 106/264 | 1.25 (0.99-1.58) | 0.065 | 0.441 |  | 325/97 | 156/54 | 1.18 (0.85-1.63) | 0.333 | 0.410 |
| Histological type | Squamous cell carcinoma | 42/96 | 35/78 | 1.63 (1.00-2.65) | 0.05 |  |  | 112/26 | 45/17 | 1.42 (0.77-2.62) | 0.261 |  |
|  | Adenocarcinoma | 139/339 | 116/298 | 1.19 (0.96-1.49) | 0.112 |  |  | 365/113 | 165/63 | 1.33 (0.99-1.80) | 0.059 |  |
|  | Other^d^ | 16/49 | 13/44 | 2.08 (0.93-4.66) | 0.076 |  |  | 49/16 | 24/9 | 1.14 (0.42-3.12) | 0.793 |  |
|  | NSCLC-NOS | 10/19 | 5/15 | 2.15 (0.26-17.87) | 0.480 | 0.405 |  | 21/8 | 6/3 | N/A |  | 0.937 |
| Histologic grade | Poorly differentiated | 125/290 | 98/257 | **1.38 (1.08-1.75)** | **0.009** |  |  | 318/97 | 143/56 | 1.27 (0.92-1.75) | 0.143 |  |
|  | Well-  moderately differentiated | 82/213 | 71/178 | 1.30 (0.97-1.75) | 0.076 | 0.799 |  | 229/66 | 97/36 | **1.55 (1.03-2.34)** | **0.035** | 0.447 |
| Chemotherapy regimen 1 | Platinum-  docetaxel/  paclitaxel | 65/172 | 54/148 | **1.71 (1.20-2.44)** | **0.003** |  |  | 180/57 | 71/30 | 1.30 (0.82-2.06) | 0.271 |  |
|  | Platinum-  etoposide | 1/6 | 0/5 | **N/A** |  |  |  | 5/2 | 1/0 | N/A |  |  |
|  | Platinum-  gemcitabine | 40/92 | 34/85 | 0.96 (0.62-1.49) | 0.867 |  |  | 103/29 | 55/20 | 1.21 (0.70-2.12) | 0.497 |  |
|  | Platinum-  vinorelbine | 8/26 | 8/22 | 1.08 (0.34-3.45) | 0.899 |  |  | 28/6 | 11/3 | 0.02 (0-1.23) | 0.063 |  |
|  | Platinum-  pemetrexed | 93/207 | 73/175 | 1.07 (0.80-1.42) | 0.664 | 0.133 |  | 231/69 | 102/39 | 1.38 (0.94-2.02) | 0.103 | 0.356 |
| Chemotherapy regimen 2 | Cisplatin-  combinations | 165/408 | 133/353 | **1.24 (1.01-1.52)** | **0.044** |  |  | 444/129 | 202/70 | 1.19 (0.90-1.57) | 0.221 |  |
|  | Carboplatin-  combinations | 42/95 | 36/82 | **1.59 (1.02-2.49)** | **0.040** | 0.311 |  | 103/34 | 38/22 | **2.30 (1.24-4.24)** | **0.008** | 0.056 |
| Grade 3/4 chemotherapy toxicity | No | 139/326 | 115/279 | **1.46 (1.16-1.83)** | **0.001** |  |  | 372/93 | 166/54 | 1.18 (0.86-1.63) | 0.301 |  |
|  | Yes | 68/177 | 54/156 | 1.12 (0.80-1.56) | 0.516 | 0.199 |  | 175/70 | 74/38 | **1.74 (1.13-2.69)** | **0.012** | 0.158 |
| Palliative radiotherapy | No | 142/349 | 122/308 | **1.32 (1.06-1.64)** | **0.013** |  |  | 378/113 | 182/64 | 1.28 (0.94-1.72) | 0.114 |  |
|  | Yes | 65/154 | 47/127 | 1.19 (0.83-1.71) | 0.341 | 0.638 |  | 169/50 | 58/28 | 1.53 (0.91-2.58) | 0.107 | 0.549 |
| TKI treatment | No |  |  |  |  |  |  | 356/97 | 155/53 | 1.25 (0.91-1.73) | 0.172 |  |
|  | Yes |  |  |  |  |  |  | 191/66 | 85/39 | **1.63 (1.09-2.46)** | **0.018** | 0.316 |
| **Supplemental Table S5. Stratified multivariate analysis of clinical variables for associations between NER risk SNPs and PFS and OS in patients with advanced NSCLS.** ^a^Adjusted by age at treatment, sex, smoking status, TNM stage, histological type, histologic grade, ECOG performance status, chemotherapy regimens, grade 3/4 chemotherapy toxicity and palliative radiotherapy (the stratified factor in each stratum excluded). ^b^Adjusted by age at treatment, sex, smoking status, TNM stage, histological type, histologic grade, ECOG performance status, chemotherapy regimens, grade 3/4 chemotherapy toxicity, palliative radiotherapy and tyrosine-kinase inhibitor treatment (the stratified factor in each stratum was excluded). ^c^ Patients were categorized as never smokers ( < 100 lifetime cigarettes), former smokers (quit ≥ 1 year ago), or current smokers (quit < 1 year ago) at the time of diagnosis. The results are in **bold**, if *P*< 0.05. | | | | | | | | | | | | |
| Abbreviations: NER, nucleotide excision repair; SNP, single nucleotide polymorphism; PFS, progression free survival; OS, overall survival; NSCLC, non-small cell lung cancer; LRi, low risk group; HRi, high risk group; HR, hazards ratio; CI, confidence interval; hom, heterogeneity test; TKI, tyrosine kinase inhibitor. | | | | | | | | | | | | |

| **SNP ID** | **primer_F** | **primer_R** | **chr** | **pos1** | **pos2** | **pos3** | **pos4** | **length** |
| --- | --- | --- | --- | --- | --- | --- | --- | --- |
| rs3212986 | AGTGCCCCAAGAGGAGATGCCAG | TCCTCCCAGGCCAGGCTCC | 19 | 45912651 | 45912673 | 45912871 | 45912889 | 239 |
| rs2298881 | TACAGGTCCACAAGTCCCATCG | TTGAGGCTCCAAGACCAGCA | 19 | 45926832 | 45926853 | 45927082 | 45927101 | 270 |
| rs3212924 | CCCGTGTTGTCCTCCCAGAGCTTAC | TGCTTGAATGTGGGTGGCGGA | 19 | 45928533 | 45928557 | 45928730 | 45928750 | 218 |
| rs3212930 | GGTCCTGTGCGAGTCCGTGCT | CTCATCAGCCAGGCCCCTGC | 19 | 45927522 | 45927542 | 45927750 | 45927769 | 248 |
| rs50871 | TGAGAACCTAAGGATGAGGGGGAGGTG | AGATCTGGGCCCTTGCCCTGG | 19 | 45862444 | 45862470 | 45862635 | 45862655 | 212 |
| rs3916788 | TCTGGGCCCAAGAGTTAGAAATG | GGCGCCATCTCTGCTCAC | 19 | 45874274 | 45874296 | 45874528 | 45874545 | 272 |
| rs238416 | GACCACTGGGACAGAATGGAGA | AGACACCTTTGCCTACCCCAA | 19 | 45856962 | 45856983 | 45857190 | 45857210 | 249 |
| rs2097215 | CATAGTGGCATGCATCTGTAATCC | GCTCTACTCAAACCTCGCTTCTTC | 19 | 45875626 | 45875649 | 45875855 | 45875878 | 253 |
| rs4150477 | CTTACAAGCTGCCAGGCACAA | GAGAGGCTTAGGGTTATATGGCAGT | 2 | 128032455 | 128032475 | 128032632 | 128032656 | 202 |
| rs13385611 | TGTGGCAGCATTTCACAAAACTAC | TCCACTGTTGGTGGATGATGTAGA | 2 | 128004605 | 128004628 | 128004846 | 128004869 | 265 |
| rs3738948 | CATGGCATTGTGCCTTTTTGT | GGAGGACAGGGTTGTCTGACC | 2 | 128017843 | 128017863 | 128018093 | 128018113 | 271 |
| rs1799798 | CACTGCCACCCAGCCTGCC | CGCAGTGTGAGGGACCTGCATC | 16 | 14014176 | 14014194 | 14014389 | 14014410 | 235 |
| rs3136038 | CCCTCTAGTGGAAGGAGGGATTT | CCTGTTTCCTAGGGTGGTTTTTG | 16 | 14013302 | 14013324 | 14013503 | 14013525 | 224 |
| rs751402  &rs2296147 | CGCGTCGTATTAGACGGAAACC | GACTCTGGCTGCAACACGTCTC | 13 | 103498159 | 103498180 | 103498407 | 103498428 | 270 |
| rs2094258  &rs3759497 | AAAATCAGGGCGTGGTGGT | TTGGAACGAAATTTAAACTCAGTGAA | 13 | 103496534 | 103496552 | 103496781 | 103496806 | 273 |
| rs873601 | ATCCTCTATAATTAGTTATGACAGCCATTTG | ACCAGGAAGGATCAGACATTGTTAAG | 13 | 103528267 | 103528297 | 103528499 | 103528524 | 258 |
| rs1982546 | CAGCCAAGTATCACCTTCATTTGC | AAGATGCACCCAAGTTTATTCTGCTA | 3 | 14217216 | 14217239 | 14217438 | 14217463 | 248 |
| rs2229090 | CCTGCAGCACCTCCTCAGC | AAGGGTGGGCCCAAGAAGA | 3 | 14187261 | 14187279 | 14187497 | 14187515 | 255 |
| rs2607772 | GAAGGACCACCTTAGTTTGCTCAAG | CTCTCCTGTTTCTCTTACCACTTGTTTC | 3 | 14218881 | 14218905 | 14219068 | 14219095 | 215 |
| rs2607775 | GGGCCTCGCTCTCACCCTCCTC | CCTCCAACGAAGGGGCGTGG | 3 | 14219951 | 14219972 | 14220160 | 14220179 | 229 |
| rs1800975 | CAAACCTCCAGTAGCCGCAGCC | CAGTGCGCGTGCGTGGAG | 9 | 100459398 | 100459419 | 100459606 | 100459623 | 226 |
| rs3176623 | AAACTAGGCTGGGCACAGTGG | GGTTCTTGTTACATTGCCCAGGT | 9 | 100460806 | 100460826 | 100461036 | 100461058 | 253 |
| rs7041137 | TACTGCTGAGGATCCAGAAGCAC | CAACTTCCTATCTCTGTTATCCTGCTCT | 9 | 110044524 | 110044546 | 110044735 | 110044762 | 239 |

**Supplemental Table S6. Characteristics of 25 pairs primers and amplicons used in sequencing.**

Abbreviations: SNP, single nucleotide polymorphism; primer_F, forward primer; primer_R, reverse primer; chr, chromosome; pos1, forward primer 5’UTR position; pos2, forward primer 3’UTR position; pos3, reverse primer 5’UTR position; pos4, reverse primer 3’UTR position.

| **A** | **B** |
| --- | --- |
| **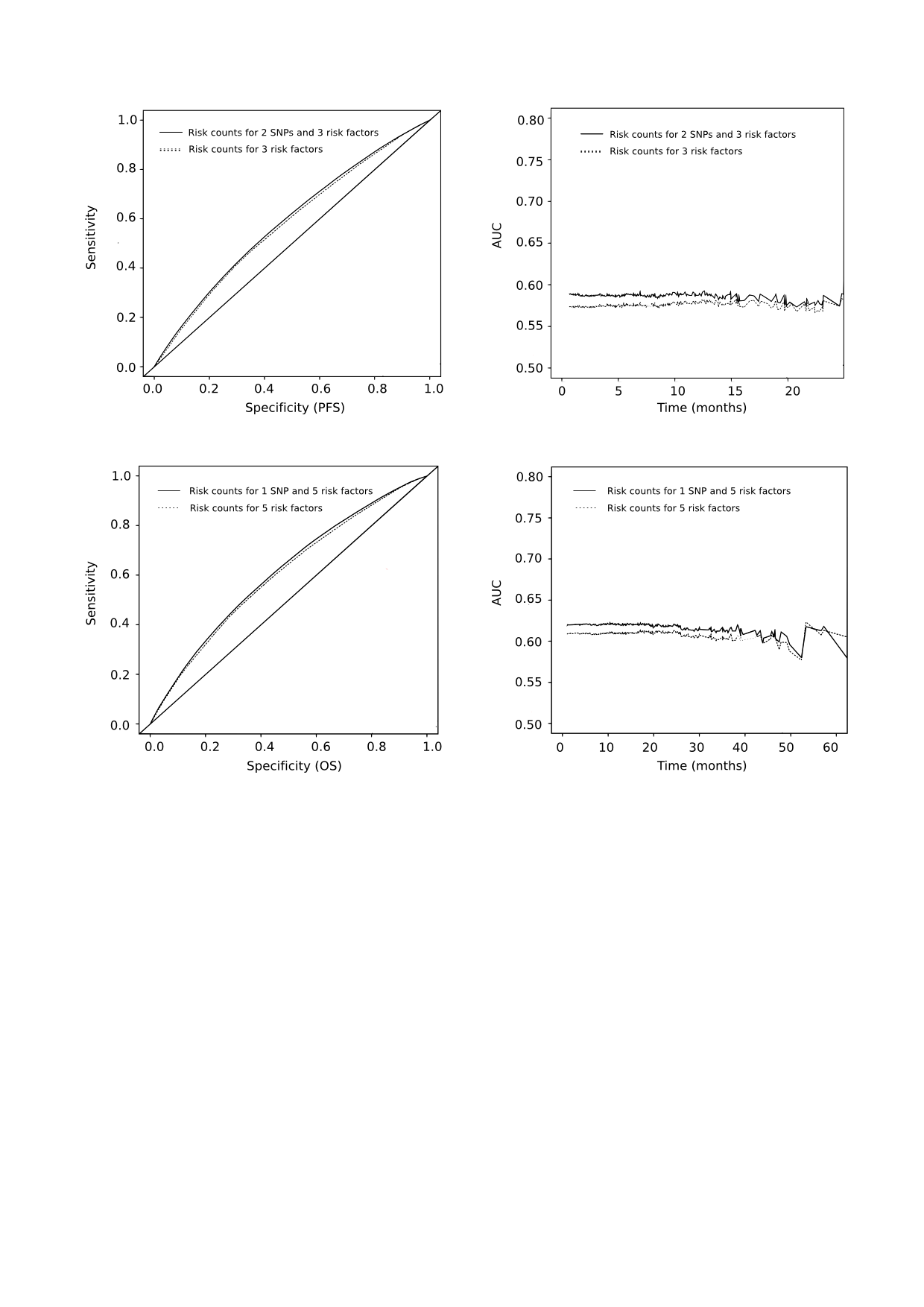** | 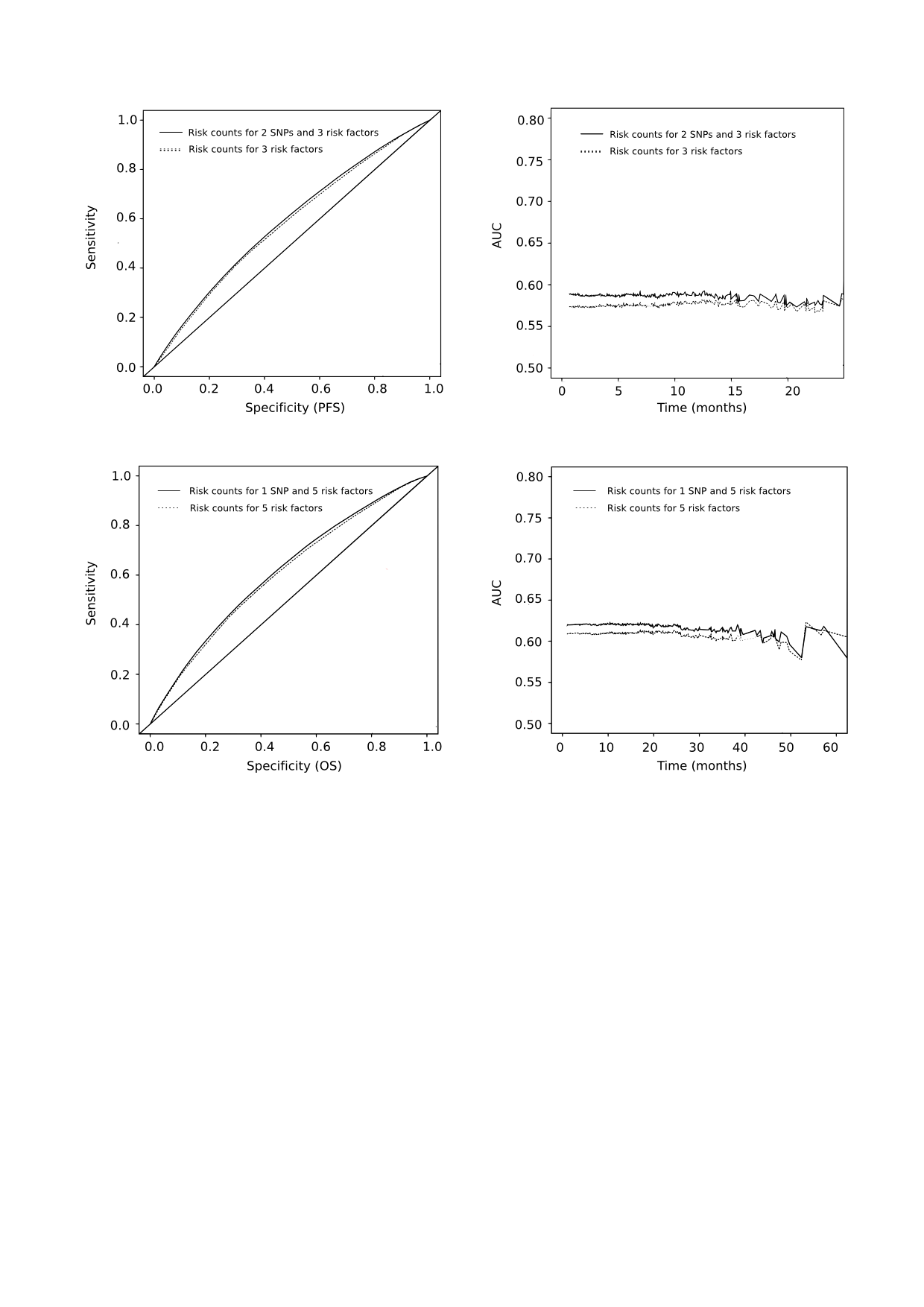 |
| **C** | **D** |
| **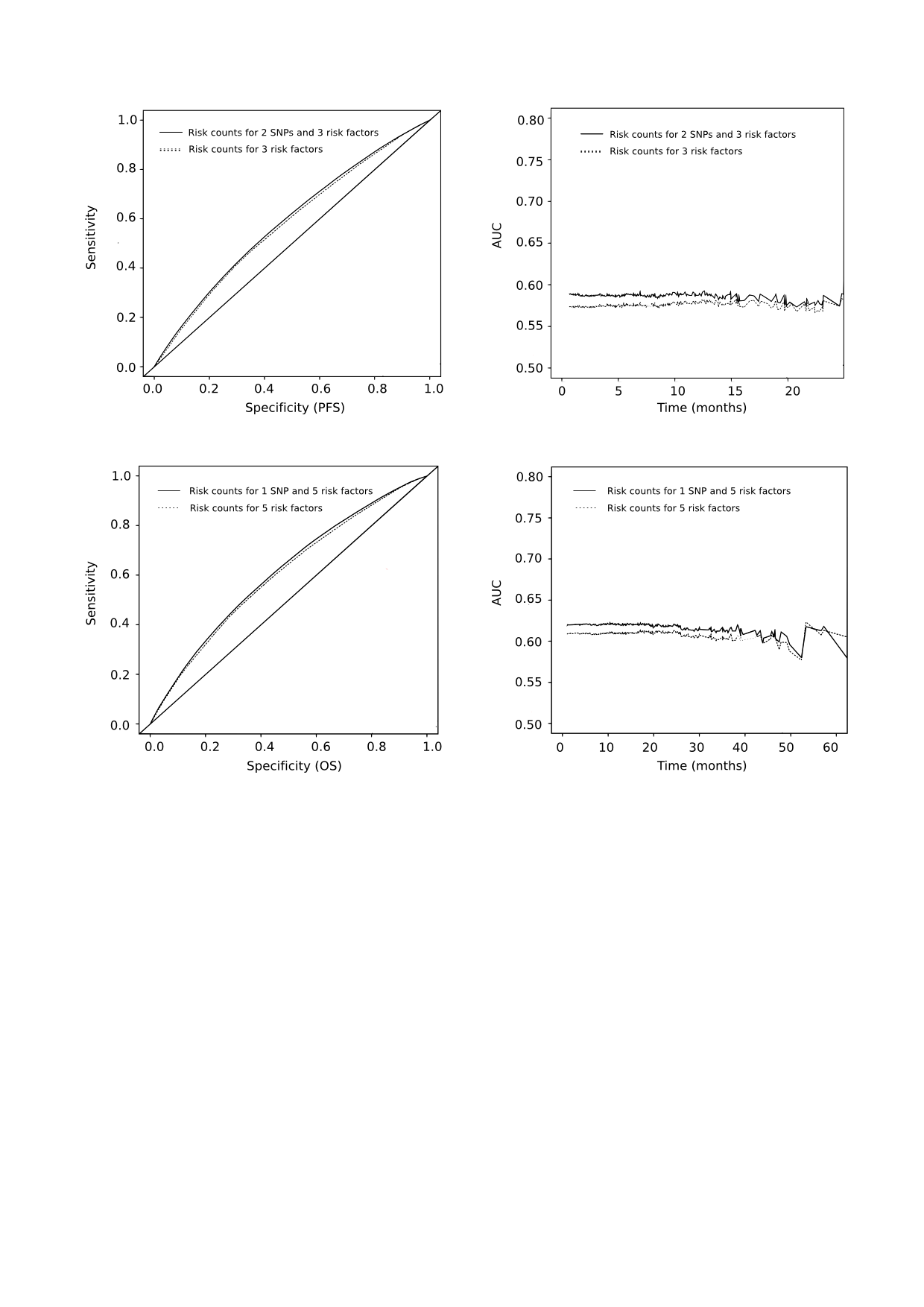** | 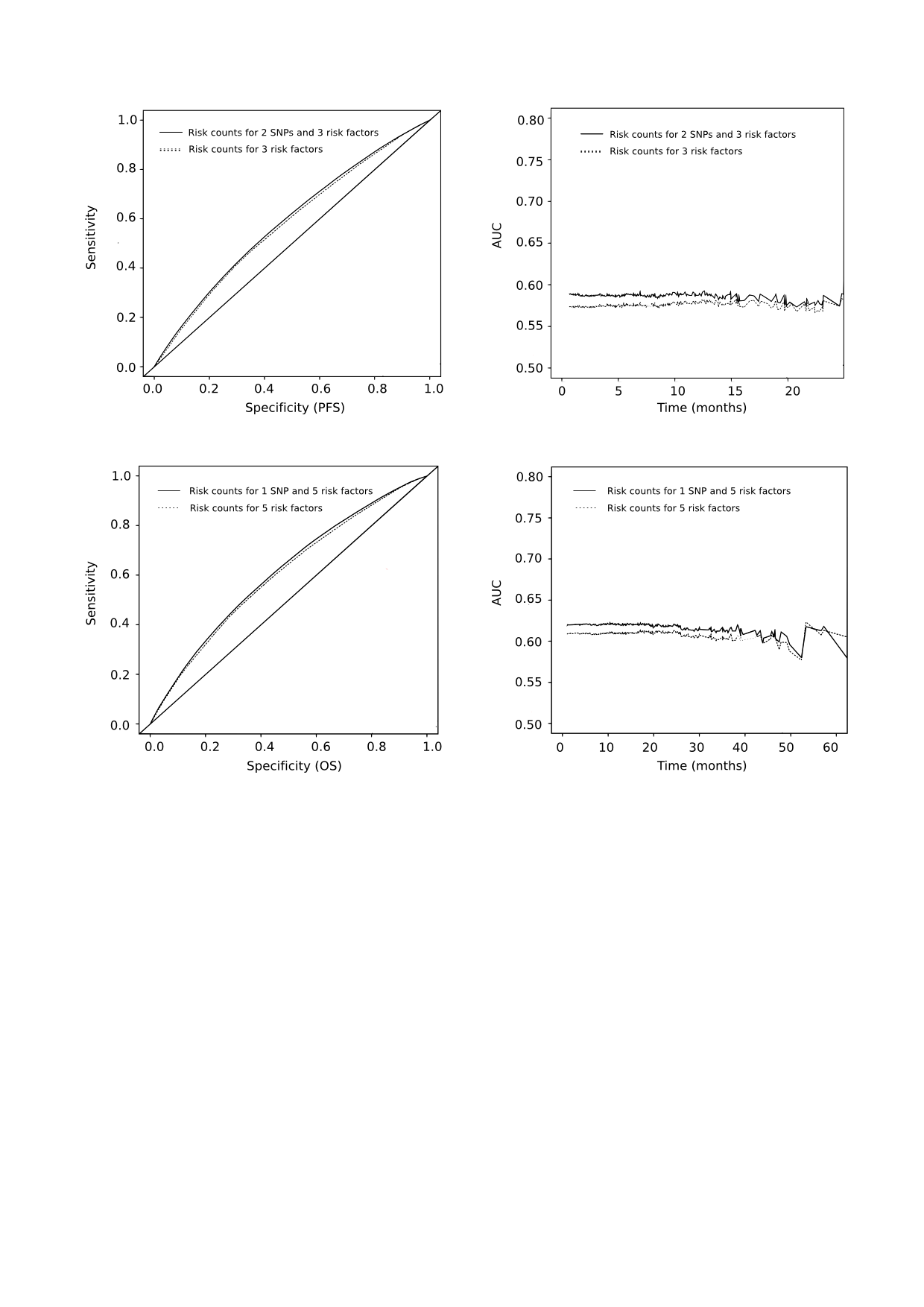 |
| **Supplemental Figure S1. Time-dependent Receiver operating characteristic (ROC) curves and incident/dynamic area under the curve (I/CAUC) plots for the prediction of progression free survival (PFS) and overall survival (OS).** (**A**) and (**B**) ROC and I/C AUC plot for PFS prediction based on combined variables including nucleotide excision repair (NER) risk genotypes (rs3212924 AG/GG and rs2229090 GG) and clinical risk factors (age, sex, ECOG and radiotherapy) or clinical risk factors only; (**C**) and (**D**) ROC and I/C AUC plot for OS prediction, based on combined variables including NER risk genotypes (rs1799798 GA/AA) and clinical risk factors (age, sex, smoking status, radiotherapy and TKI therapy) or clinical risk factors only. | |

|  | **OS** | | |  | **PFS** | | |
| --- | --- | --- | --- | --- | --- | --- | --- |
|  | Clinical variables + rs1799798 | Clinical variables | *P* |  | Clinical variables + SNPs | Clinical variables | *P* |
| AUC of 1-year ROC curve (95%CI) | 0.62 (0.58-0.64) | 0.61 (0.58-0.64) | 0.374 | AUC of 1-year ROC curve (95%CI) | 0.59 (0.56-0.61) | 0.58 (0.55-0.60) | 0.295 |
| AUC of 3-year ROC curve (95%CI) | 0.61 (0.58-0.64) | 0.60 (0.57-0.63) | 0.376 | *C* index (95%CI) | **0.59 (0.56-0.61)** | **0.58 (0.55-0.59)** | **0.019** |
| AUC of 5-year ROC curve (95%CI) | 0.58 (0.54-0.64) | 0.58 (0.53-0.64) | 0.466 |  |  |  |  |
| *C* index (95%CI) | 0.61 (0.58-0.64) | 0.61  (0.58-0.63) | 0.111 |  |  |  |  |
| **Supplemental Table S7. Comparisons of AUCs of the time-dependent ROC curves and estimated C index for patients with NSCLC.**  Abbreviations: OS, overall survival; PFS, progression free survival; NSCLC, non-small cell lung cancer; AUC, area under the curve; ROC, receiver operation characteristic. | | | | | | | |
